# Distribution and prevalence of fungemia: a five-year retrospective multicentric survey in Venetian region, Italy

**DOI:** 10.1093/jacamr/dlaf044

**Published:** 2025-03-25

**Authors:** Nicholas Geremia, Beatrice Bragato, Federico Giovagnorio, Gianluca Zuglian, Pierluigi Brugnaro, Maria Solinas, Paola Stano, Sandro Panese, Saverio Giuseppe Parisi

**Affiliations:** Department of Clinical Medicine, Unit of Infectious Diseases, Ospedale ‘dell’Angelo’, Venice 30174, Italy; Department of Clinical Medicine, Unit of Infectious Diseases, Ospedale Civile ‘S.S. Giovanni e Paolo’, Venice 30122, Italy; Department of Molecular Medicine, University of Padua, Padua 35121, Italy; Department of Molecular Medicine, University of Padua, Padua 35121, Italy; Department of Clinical Medicine, Unit of Infectious Diseases, Ospedale ‘dell’Angelo’, Venice 30174, Italy; Department of Clinical Medicine, Unit of Infectious Diseases, Ospedale Civile ‘S.S. Giovanni e Paolo’, Venice 30122, Italy; Department of Clinical Medicine, Unit of Infectious Diseases, Ospedale Civile ‘S.S. Giovanni e Paolo’, Venice 30122, Italy; Department of Medical direction, Unit of Microbiology and Virology, Ospedale ‘Dell’Angelo’, Venice 30174, Italy; Department of Medical direction, Unit of Microbiology and Virology, Ospedale ‘Dell’Angelo’, Venice 30174, Italy; Department of Clinical Medicine, Unit of Infectious Diseases, Ospedale ‘dell’Angelo’, Venice 30174, Italy; Department of Clinical Medicine, Unit of Infectious Diseases, Ospedale Civile ‘S.S. Giovanni e Paolo’, Venice 30122, Italy; Department of Molecular Medicine, University of Padua, Padua 35121, Italy

## Abstract

**Background:**

Invasive fungal infections, significantly impact hospitalized and immunocompromised populations. Recent trends showed a shift from *Candida albicans* to non-*albicans Candida* (NAC) species, raising concerns about antifungal resistance.

**Objectives:**

Our study focuses on the distribution of fungal species in blood cultures obtained from different healthcare settings, including hospitals, long-term care facilities, and community health centers in the Venetian region of Italy.

**Methods:**

We retrospectively analyzed all consecutive blood culture isolates across 5 hospitals, 38 long-term care facilities, and 24 sample collection centers (blood exams and culture) from 2019 to 2023.

**Results:**

Between 2019 and 2023, 11,552 microorganisms were isolated from blood cultures; 693 (6.0%) were fungi. The yearly prevalence ranged from 5.2% in 2019 to 6.1% in 2023. *C. albicans* isolates decreased significantly, from 60.0% in 2019 to 43.1% in 2023. NAC species showed significant growth, particularly *C. parapsilosis sensu stricto* (from 23.6% in 2019 to 28.8% in 2023), *C. tropicalis* (from 0.0% in 2019 to 7.2% in 2023), and *N. glabratus* (from 9.1% in 2019 to 11.8% in 2023). Medical wards consistently recorded the highest number of cases (429/693, 61.9%), with *C. albicans* predominating in earlier years. Resistance to amphotericin B rose sharply in *C. parapsilosis* ss. (22.5% in 2022), while fluconazole resistance in *N. glabratus* remained high (peaking at 85.7% in 2021).

**Conclusion:**

The increasing dominance of NAC species and rising resistance trends underscore the necessity for enhanced diagnostics, infection prevention, and antifungal stewardship. Future research should incorporate clinical data to optimize fungemia management strategies.

## Introduction

Invasive fungal infections (IFIs) represent a major global health concern, particularly in hospitalized and immunocompromised populations.^1^ These infections contribute significantly to morbidity and mortality, with an estimated 1.5 million deaths annually. Over 90% of these fatalities are attributed to four primary genera: *Cryptococcus*, *Candida*, *Aspergillus*, and *Pneumocystis.*^2,3^

Among these, *Candida* species are the leading cause of IFIs. Traditionally, the most frequently affected patients were the critically ill patients in intensive care units (ICUs), particularly in individuals undergoing intensive medical treatments, such as invasive procedures, prolonged stays, abdominal surgery, neutropenia or treatment for major trauma.^4,5^ However, there have been recent reports of a sharp increase in candidemia among patients admitted to internal medicine and geriatric wards, probably due to the rise of patients with a central venous catheter, peripheral parenteral nutrition (PPN), total parenteral nutrition (TPN), urinary catheters, comorbidities (such as diabetes mellitus, liver diseases, tumors and malnutrition), and under broad-spectrum or unnecessary prolonged antibiotic therapy.^6,7^

*Candida albicans* is humans’ most commonly identified fungal pathogen.^8^ However, non-*albicans Candida* (NAC) species, including *Nakaseomyces glabratus*, *C. tropicalis*, and *C. parapsilosis*, have shown an alarming increase in prevalence, surpassing *C. albicans* in some settings.^9^ At the same time, emerging and uncommon *Candida* spp., such as *C. auris*, have been reported as an increasing cause of invasive *Candida* infections worldwide, determining a preoccupant trend in fungal resistance and problems in therapeutic management in IFIs.^10,11^

It is known that difficulties with *Candida* species identification, especially the emergence of new species and the lack of awareness, have resulted in transmission and several outbreaks worldwide.^12^ In particular, rapid diagnosis and antifungal susceptibility testing represent significant issues in *Candida* infection management.^13^ Therapeutic options for IFIs are limited to polyenes, 5-fluorocytosine, azoles and echinocandins. While polyenes and echinocandins are generally effective, agricultural use of azole pesticides has contributed to resistance in environmental and clinical yeasts and molds.^3,10^ Furthermore, antifungal treatments can be particularly challenging in the case of NAC, where a high azole-resistant rate is demonstrated, particularly in the case of *N. glabratus*, *Pichia kudriavzevii* or *Meyerozyma guilliermondii.*^11,14^

This retrospective study focuses on the distribution of fungal species in blood cultures obtained from different healthcare settings, including hospitals, long-term care facilities, and community health centers in the Venetian region of Italy. Additionally, it evaluates antifungal susceptibility trends over 5 years (2019–2023), aiming to provide insights into evolving resistance patterns and their implications for clinical practice.

## Material and methods

This retrospective study collected data on all consecutive fungal isolates from blood cultures obtained between January 2019 and December 2023 in the Venetian region of Italy. The samples were derived from 5 hospitals (Ospedale SS. Giovanni e Paolo, Ospedale ‘dell’Angelo’, Ospedale di Chioggia, Ospedale di Mirano, and Ospedale di Dolo), 38 long-term care facilities, and 24 sample collection centers (where blood exams and blood culture were collected from outpatients). The Venetian territory has 624,342 inhabitants, with a population density of 458.4 inhabitants/km^2^. The region presents a hub hospital (Ospedale ‘dell’Angelo’) with 580 beds and ∼20 operating rooms. The center also hosts the allogeneic and autologous stem-cell transplant center (6 beds). The surgery department counts the following units: neurosurgery, cardio surgery, general surgery, urology, plastic surgery, otolaryngology, maxillofacial surgery, and gynecology. Moreover, it counts 24 beds in ICUs. There are 4 spoke hospitals: Ospedale SS. Giovanni e Paolo, Ospedale di Chioggia, Ospedale di Mirano, and Ospedale di Dolo. Ospedale SS. Giovanni e Paolo counts 358 beds, of them 8 beds in ICUs. Ospedale di Chioggia has 150 beds with 6 ICU beds. Ospedale di Mirano and Ospedale di Dolo count 315 and 54 beds, respectively. ICUs in these two hospitals have 9 and 6 beds. The 38 long-term care facilities have a total of 3978 beds.

In Ospedale ‘dell’Angelo’ and Ospedale SS. Giovanni e Paolo, there are two Infectious Diseases (ID) units. At Ospedale ‘dell’Angelo’, an ID specialist has held an active consultant role in critical settings (ICUs from 2021 and the Stem-Cell Transplantation Center from 2023).

### Microbiology

Blood cultures were processed using BD Bactec systems (bioMérieux, Florence, Italy). Positive samples, with fungal cells visible on Gram stain examination, were subcultured on Sabouraud Dextrose Emmons Agar (SGC2, bioMérieux Inc.). For *Candida auris* isolates, Brilliance^™^  *Candida* Agar Base (Thermo Fisher Scientific^™^, Waltham, MA, USA) was employed. We also used chromogenic agar for subculturing blood culture vials to determine the presence of polymicrobial *Candida* species. Species identification was performed using MALDI-TOF/MS (Bruker, Bremen, Germany). Antifungal susceptibility testing was conducted using broth microdilution with MICRONAUT-AM (MERLIN Diagnostika GmbH, Bornheim, Germany). Susceptibility interpretations were based on the EUCAST breakpoints valid as of 1 January 2024.

### Definitions and exclusion criteria

Episodes of fungemia were defined as positive blood cultures for yeasts or molds. In the case of repetition of fungal isolates in the same patient within 30 days, the sample was dropped out of the study. Four settings of fungemia isolation were considered, including outpatient settings, medical wards, surgical wards and ICUs. Outpatient settings included isolates from long-term care facilities and sample collection centers.

### Statistical analysis

Data were collected with Excel (Microsoft, Redmond, WA, USA). The prevalence of fungemia was calculated as the number of fungal isolates divided by the total number of isolates from blood cultures. Discrete variables were summarized as frequency (%). The prevalence of antifungal resistance was calculated as the number of resistant isolates divided by the total number of tested isolates and expressed in percentage (%). The difference in resistance rate to different antifungal agents over the study years was tested through an association test for trends in proportions (Cochran-Arnitage test). A significant trend in antifungal resistance was considered in the case of *P*-value (*P*) < 0.05. Statistical analysis was conducted with STATA version 18.5 (StatsCorp, Lakeway, TX, USA).

### Ethics

The research was conducted in accordance with the Declaration of Helsinki and national and institutional standards. This study did not require ethical approval, based solely on microbiological surveillance data without involving patient-specific clinical data. All collected information was fully anonymized.

## Results

### Overview

Between 2019 and 2023, 11,552 microorganisms were isolated from blood cultures; 693 (6.0%) were fungi. The yearly prevalence of fungemia was 55/1056 (5.2%) in 2019, 119/2364 (5.0%) in 2020, 163/2539 (6.4%) in 2021, 203/3082 (6.6%) in 2022 and 153/2511 (6.1%) in 2023. Table 1 summarizes the fungal isolates divided per year.

**Table 1.** All 693 fungal isolates from blood culture from 2019 to 2023

Microorganism	2019 (55)	2020 (119)	2021 (163)	2022 (203)	2023 (153)	Tot. (693)
*Candida albicans*, *n*. (%)	33 (60.0)	69 (58.0)	92 (56.4)	100 (49.4)	66 (43.1)	360 (51.9)
*Candida parapsilosis sensu stricto*, *n*. (%)	13 (23.6)	25 (21.0)	37 (22.7)	42 (20.7)	44 (28.8)	161 (23.2)
*Nakaseomyces glabratus*, *n*. (%)	5 (9.1)	12 (10.1)	14 (8.6)	31 (15.3)	18 (11.8)	80 (11.5)
*Candida tropicalis*, *n*. (%)	0 (0.0)	2 (1.7)	5 (3.1)	10 (4.9)	11 (7.2)	28 (4.0)
*Candida orthopsilosis, n*. (%)	1 (1.8)	2 (1.7)	3 (1.8)	11 (5.4)	4 (2.6)	21 (3.0)
*Pichia kudriavzevii*, *n*. (%)	1 (1.8)	6 (5.0)	1 (0.6)	3 (1.5)	4 (2.6)	15 (2.2)
Other Yests^a^, *n*. (%)	1 (1.8)	0 (0.0)	4 (2.5)	6 (3.0)	3 (2.0)	14 (2.0)
*Clavispora lusitaniae*, *n*. (%)	0 (0.0)	2 (1.7)	5 (3.1)	0 (0.0)	2 (1.3)	9 (1.3)
A*spergillus nidulans*, *n*. (%)	0 (0.0)	0 (0.0)	1 (0.6)	0 (0.0)	0 (0.0)	1 (0.1)
*Cryptococcus neoformans*, *n*. (%)	1 (1.8)	0 (0.0)	0 (0.0)	0 (0.0)	0 (0.0)	1 (0.1)
*Cyberlinfner jadinii*, *n*. (%)	0 (0.0)	0 (0.0)	0 (0.0)	0 (0.0)	1 (0.7)	1 (0.1)
*Fusarium* spp., *n*. (%)	0 (0.0)	0 (0.0)	1 (0.6)	0 (0.0)	0 (0.0)	1 (0.1)
*Geotrichum capitatum*, *n*. (%)	0 (0.0)	1 (1.7)	0 (0.0)	0 (0.0)	0 (0.0)	1 (0.1)

^a^ *Candida kefyr* 4 cases, *Meyerozyma guilliermondii* 3 cases, *Candida dubliniensis* 2 cases, *Candida auris* 1 case, *Cyberlindnera fabianii* 1 case, *Candida metapsilosis* 1 case, *Candida rugosa* 1 case, *Pichia kluyveri* 1 case.

**Figure 1.**
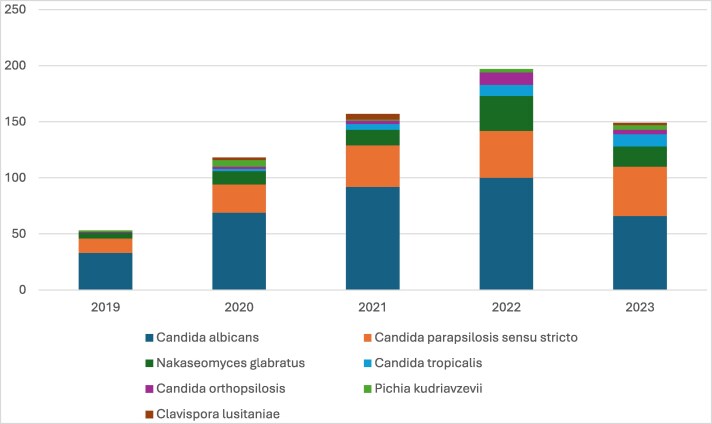
Principal *Candida* isolates from blood culture divided per year.

**Figure 2.**
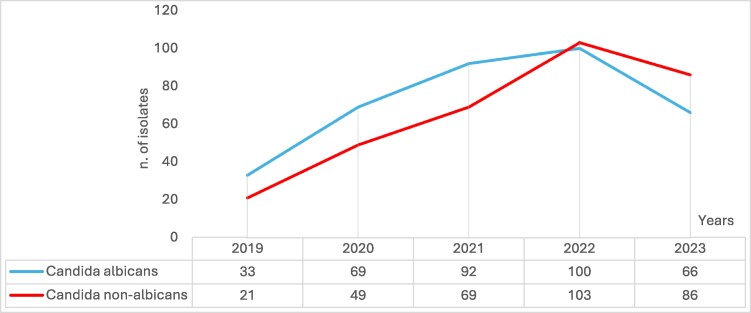
Trend of Candidemia from 2019 to 2023. In 2022 and 2023, the Non-*albicans Candidemia* had surpassed the *Candida albicans* fungemia.

*Candida* species represented the majority of fungal isolates in blood cultures (688/693. 99.3%). The most commonly identified species were *C. albicans* (360/693, 51.9%), followed by *C. parapsilosis sensu stricto* (161/639, 23.2%) and *N. glabratus* (80/693, 11.5%). Figure 1 shows the principal *Candida* isolates from blood culture divided per year. Notably, *C. albicans* isolates decreased significantly, from 60.0% in 2019 to 43.1% in 2023 (Figure 2). At the same time, there was an increase in NAC species, particularly *C. parapsilosis ss.* (from 23.6% in 2019 to 28.8% in 2023) and *C. tropicalis* (from 0.0% in 2019 to 7.2% in 2023). *N. glabratus* also showed a moderate increase, rising from 9.1% in 2019 to 11.8% in 2023.

### Distribution by setting

**Figure 3.**
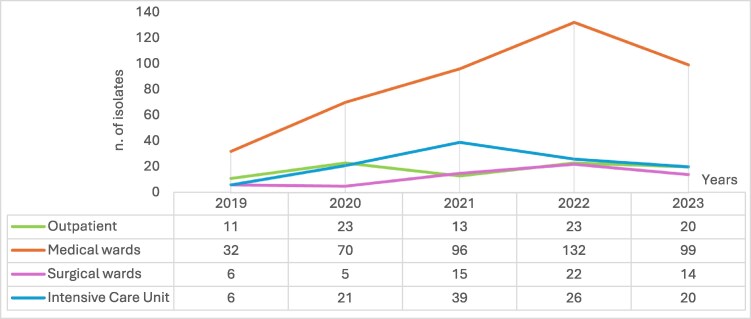
Number of fungemia from different settings per year.

Medical wards consistently recorded the highest number of cases (429/693, 61.9%), with *C. albicans* predominating in earlier years (225 cases, 52.4%). However, in 2023, NAC species collectively accounted for 54 cases of *Candida* isolates (54.6%), led by *C. parapsilosis ss.* (27 isolates, 27.3%) and *N. glabratus* (12 isolates, 12.1%). Surgical wards showed a less dramatic but steady presence of fungemia (62/639, 9.7%), primarily driven by *C. albicans* (36 cases, 58.1%) and secondly by *C. parapsilosis ss.* (17 cases, 27.4%). The fungemia trend from different settings per year is summarized in Figure 3. In ICUs, the number of yeast isolates was 112/693 (16.2%), with a reduction in candidemia cases from 2021 to 2023. *C. albicans* cases were 61 (54.5%) from 2019 to 2023. However, a decreased trend was observed from 2021 (20 cases) to 2023 (10 cases).

**Figure 4.**
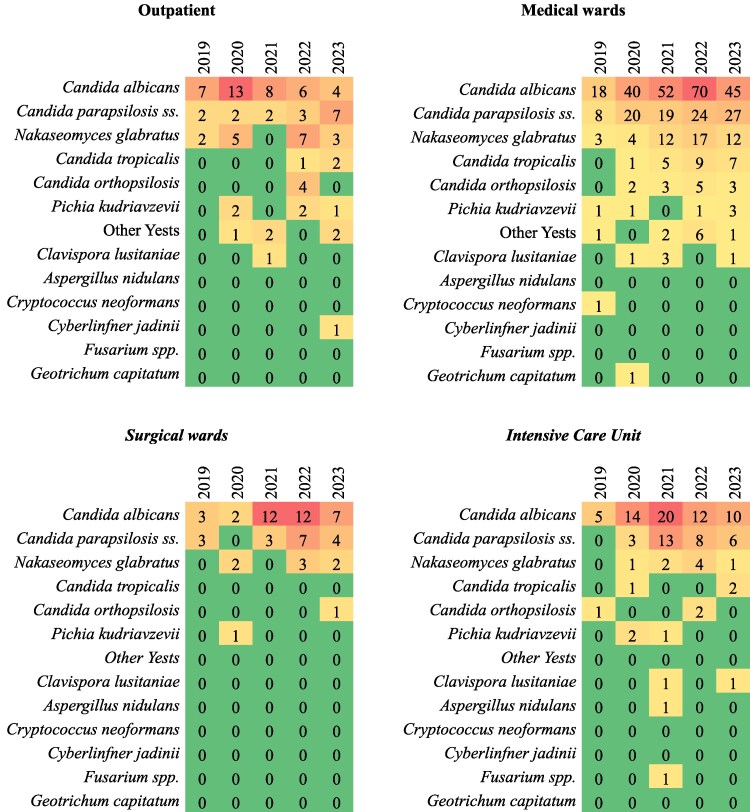
Fungi isolates from different settings.

Outpatient cases were limited in number (90/69 313.0%) but revealed a gradual diversification of isolates. Forty-seven (52.2%) patients were from long-term facilities, and 43 cases (47.8%) were collected from outpatient sample collection centers. Most cases were related to *C. albicans* (38 isolates, 42.2%). NAC has been rising in that setting, particularly in the case of *N. glabratus* (17 cases, 18.8%) and *C. parapsilosis ss.* (16 cases, 17.8%). Figure 4 shows fungal isolates from blood cultures in different settings.

### Trends in antifungal resistance

#### Candida albicans

No significant resistance trend of amphotericin B (AMB) was observed, with a consistent susceptibility over the study period. Echinocandin resistance was rare, with an initial rate of 6.1% in 2019 that decreased to 0.0% by 2023 (*P* < 0.0001). Resistance to azoles was minimal, with isolated cases of fluconazole (FLU) resistance (2.4% in 2021, 1.5% in 2023).

#### Candida parapsilosis sensu stricto

A significant increase in AMB resistance was observed, rising from 4.3% in 2020 to 22.5% in 2022 (*P* = 0.003). Echinocandin resistance was rare (2.3% in 2023), with no statistically significant trend (*P* = 0.75). FLU resistance was variable during the observational period, peaking at 13.9% in 2021 and stabilising at 9.1% in 2023. Posaconazole resistance was negligible.

#### *Nakaseomyces glabratus* and other species

**Figure 5.**
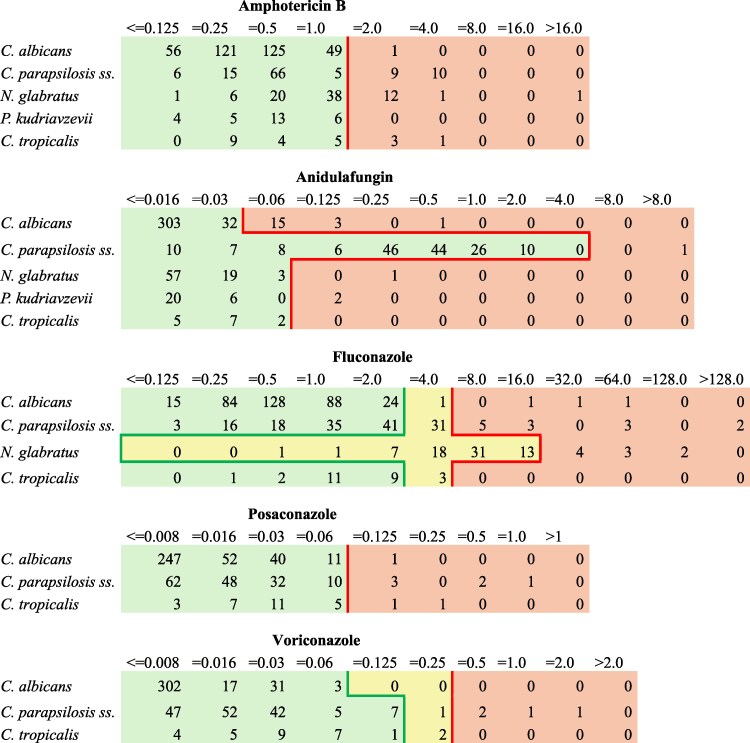
MICs (mg/L) distribution per antifungal and species. The red line refers to the Breakpoint; instead, the green line refers to the susceptibility cut-off. In light green susceptible isolates, light yellow intermediate isolates and light red resistant isolates. Susceptibility interpretations were based on the EUCAST breakpoints valid as of 1 January 2024.

FLU resistance was notably high, peaking at 85.7% in 2021 and decreasing to 55.6% in 2023 (*P* = 0.08). No other antifungal resistance trends were seen. For *C. tropicalis* and *P. kudriavzevii,* rare cases of resistance were observed occasionally. Table 2 shows trends in antifungal resistance, and Figure 5 shows the MIC distribution.

**Table 2.** Trend in antifungal resistance of the principal *Candida* isolates during 2019–2023

Microorganism	2019 (%)	2020 (%)	2021 (%)	2022 (%)	2023 (%)	*P* for trend^a^	Resistance rate total (%)
**Amphotericin B**							
*C. albicans*	0/33 (0.0)	0/62 (0.0)	1/92 (1.1)	0/99 (0.0)	0/66 (0.0)	0.43	1/352 (0.0)
*C. parapsilosis ss.*	0/13 (0.0)	1/23 (4.3)	8/37 (21.6)	9/40 (22.5)	1/42 (2.4)	0.003	19/155 (0.1)
*N. glabratus*	0/5 (0.0)	0/12 (0.0)	4/14 (28.6)	8/30 (26.7)	2/18 (11.1)	0.12	14/79 (0.2)
*P. kudriavzevii*	0/0 (0.0)	2/4 (50.0)	0/1 (0.0)	1/3 (33.3)	1/4 (25.0)	0.66	4/13 (0.3)
*C. tropicalis*		0/2 (0.0)	0/5 (0.0)	0/10 (0.0)	0/11 (0.0)		0/28 (0.0)
**Anidulafungin**							
*C. albicans*	2/33 (6.1)	15/93 (23.8)	1/92 (1.1)	1/100 (1.0)	0/66 (0.0)	<0.0001	19/354 (0.1)
*C. parapsilosis ss.*	0/13 (0.0)	0/23 (0.0)	0/37 (0.0)	0/41 (0.0)	1/44 (2.3)	0.75	1/158 (0.0)
*N. glabratus*	0/5 (0.0)	1/12 (8.3)	0/14 (0.0)	0/31 (0.0)	0/18 (0.0)	0.27	1/80 (0.0)
*P. kudriavzevii*	0/1 (0.0)	0/5 (0.0)	0/1 (0.0)	0/3 (0.0)	0/4 (25.0)	/	0/14 (0.0)
*C. tropicalis*	0/0 (0.0)	0/2 (0.0)	0/5 (0.0)	2/10 (20.0)	0/11 (0.0)	0.15	2/28 (0.1)
**Fluconazole**							
*C. albicans*	0/33 (0.0)	0/60 (0.0)	2/85 (2.4)	0/100 (0.0)	1/65 (1.5)	0.27	3/343 (0.0)
*C. parapsilosis ss.*	0/13 (0.0)	2/23 (8.7)	5/36 (13.9)	2/41 (4.9)	4/44 (9.1)	0.36	13/157 (0.1)
*N. glabratus*	2/5 (40.0)	6/12 (50.0)	12/14 (85.7)	23/31 (74.2)	10/18 (55.6)	0.08	53/80 (0.7)
*C. tropicalis*	0/0 (0.0)	0/2 (0.0)	0/3 (0.0)	0/10 (0.0)	0/11 (0.0)	/	0/26 (0.0)
**Posaconazole**							
*C. albicans*	0/33 (0.0)	1/62 (1.6)	0/91 (0.0)	0/100 (0.0)	0/65 (0.0)	0.31	1/351 (0.0)
*C. parapsilosis ss.*	0/13 (0.0)	1/23 (4.3)	1/37 (2.7)	0/41 (0.0)	0/44 (0.0)	0.51	2/158 (0.0)
*C. tropicalis*	0/0 (0.0)	1/2 (50.0)	0/5 (0.0)	1/10 (10.0)	0/11 (0.0)	0.14	2/28 (0.1)
**Voriconazole**							
*C. albicans*	0/33 (0.0)	0/63 (0.0)	0/91 (0.0)	0/100 (0.0)	0/66 (0.0)	/	0/352 (0.0)
*C. parapsilosis ss.*	0/13 (0.0)	0/23 (0.0)	1/37 (2.7)	0/41 (0.0)	3/41 (6.8)	0.43	4/158 (0.0)
*C. tropicalis*	0/0 (0.0)	0/2 (0.0)	0/5 (0.0)	0/10 (0.0)	0/11 (0.0)	/	0/28 (0.0)

^a^Cochran-Arnitage test for trend in proportions, *P* < 0.05 for significant trend in antifungal resistance.

## Discussion

This 5-year retrospective study underscores a notable shift in the epidemiology of fungemia within the Venetian healthcare region, marked by the increasing dominance of NAC species over *C. albicans* and a concentration of cases in medical wards.

While *C. albicans* accounted for over half of the isolates (51.9%), its prevalence has steadily declined from 60.0% in 2019 to 43.1% in 2023. This reduction coincided with a significant rise in NAC species such as *C. parapsilosis ss.*, *N. glabratus*, and *C. tropicalis*. In 2023, NAC species collectively surpassed *C. albicans*, reflecting a paradigm shift in fungal pathogen distribution. For instance, *C. parapsilosis ss.*, which constituted 23.2% of isolates overall, increased from 23.3% in 2019 to 28.8% in 2023, while *C. tropicalis* emerged as a notable contributor, growing from 0.0% in 2019 to 7.2% in 2023. This trend is confirmed in various studies conducted not only in Europe but also in other countries. Hoenigl *et al.*^15^ managed a European observational cohort study collecting cases of candidemia from 64 institutions in 20 European countries. Of the 632 cases of candidemia, 274 cases (43.4%) collected were caused by *C. albicans*, whereas the others were NAC. Interestingly, the two most commonly isolated NAC species were *N. glabratus* and *C. parapsilosis.*^15^ Sano *et al.*^16^ conducted a similar study at the Kyorin University Hospital in Tokyo, Japan, collecting candidemia cases in their hospital center. Of the 206 strains of *Candida* isolated, 102 (49.5%) were *C. albicans*, whereas the most common NAC species were *N. glabratus* (40 strains, 19.4%) and *C. parapsilosis* (38 strains, 18.4%).^16^ Although the distribution of NAC species varies depending on geographic provenience, there could be present center-to-center and unit-to-unit variability.^17^ In the USA. and northwestern Europe, the most frequent NAC species is generally *N. glabratus* in the non-outbreak setting, and it is more common in older people and recipients of solid organ transplants.^9,18^ In Latin America, Southern Europe, and Asia, *C. parapsilosis* and/or *C. tropicalis* are much more prevalent NAC species. Often found in patients with hematological malignancies who have received antifungal prophylaxis with azoles.^9^

Outpatient candidemia is not always a defined entity, and the driving force behind the emergence of candidemia in that setting is unknown.^19^ The increase in patients’ exposure to traditional risk factors in long-term facilities and the elderly could potentially be factors that explain this phenomenon. In community settings, some authors found a prevalence of *C. albicans.*^19^ However, other species observed varied from *N. glabratus*, *C. parapsilosis* and *C. tropicalis.*^19,20^ Outpatient cases were limited in our cohort, with a high prevalence of *C. albicans* infections (42.2%). Interestingly, *N. glabratus* (18.8%) and *C. parapsilosis ss.* (17.8%) were the 2 most common NAC species isolated in outpatients. In our cohort, long-term facilities impacted the outpatient setting we examined; half of the positive fungal territory's samples were from these structures. The intrinsic frailty of the long-term care facility residents could partially explain the prevalence of NAC species.

Most candidemia and fungemia cases occurred in medical wards, accounting for 61.9% of all isolates during the study period. This distribution emphasizes the vulnerability of hospitalized patients with prolonged stays and supports medical interventions, such as PPN or TPN, or comorbid conditions. While *C. albicans* dominated earlier years, NAC species, including *C. parapsilosis ss.* and *N. glabratus*, became increasingly prevalent in 2023. Notably, in medical wards, NAC species accounted for 54.6% of isolates in 2023, highlighting their expanding role in hospital-acquired fungal infections. ICUs, are often considered hotspots for severe fungal infections. Nevertheless, in our study, ICUs presented a smaller but significant proportion of cases, with a similar trend of rising NAC species. Interestingly, the medical wards accounted for most candidemia cases in our setting, whereas ICUs showed little but no dramatic increase over the years. This trend contrasts with literature reports, where ICUs represent the most frequent setting to develop invasive candidiasis, comprising candidemia.^21–23^ This may be explained by an ID specialist conducting daily consultations in the Ospedale ‘dell’Angelo’ and Ospedale SS Giovanni e Paolo, which have the largest ICUs in the Venetian region. This ID activity promotes preventive measures, including enhanced monitoring of high-risk patients, optimisation of antifungal therapies in terms of type and duration, and avoiding unnecessary antifungal treatments.

The rise of NAC species brings heightened resistance challenges. *C. parapsilosis ss*, for instance, demonstrated a notable increase in AMB resistance, peaking at 22.5% in 2022. Although *C. parapsilosis ss.* strains resistant to azole are increasingly reported worldwide, with a growing peak since 2019, probably due to the COVID-19 pandemic,^24^ the susceptibility to azoles remains high in our setting. Yamin *et al.*^25^ conducted a systematic review and meta-analysis regarding *C. parapsilosis* antifungal resistance, highlighting a pooled resistance rate to AMB estimated to be 1.3%.^25^ The study evidenced a significant heterogeneity among the studies included, with only one reporting a prevalence of AMB resistance of 46.9%, whereas the majority reported 0% resistance to this antifungal drug.^25^ Nevertheless, it is essential to remember that polyene resistance in *Candida* species is rare, particularly in the case of *C. albicans*, *N. glabratus* and *C. parapsilosis*. Notably, in the literature, remarkable differences in AMB resistance rate have been related to AST methods, particularly in the case of broth microdilution and Etest.^25^ The resistance mechanisms of this class are less well understood. Several hypotheses have been forwarded to explain resistance, including sterol composition modulation by mutations in genes (ERG genes) involved in the ergosterol biosynthesis and enhanced defense against oxidative damage to break down the reactive oxygen species produced under polyene exposure.^26^ The high incidence of AMB-resistant *C. parapsilosis* strains reported in our 2022 study could be explained by establishing the allogeneic stem-cell transplant center in the same year. Notably, regular ID specialist consultancy was introduced only in 2023. Moreover, in our center, AMB prophylaxis in allogenic stem-cell transplantation was usually the preferred anti-mold prophylaxis regimen. The high consumption of AMB in this setting can partially explain the rise in the prevalence of AMB-resistant strains. Another critical point in *C. parapsilosis* infections was the increased number of FLU-resistant strains worldwide.^25^ The global prevalence of FLU-resistant *C. parapsilosis* is mainly variable country by country, ranging from 0% to 100%. In general, the FLU resistance rate is estimated to be 15.2%, with a dramatic trend in the last years.^25^ FLU resistance was not particularly challenging in our setting, with the highest rate in 2021 (above 13%).

Echinocandin resistance arises from amino acid alterations in the FKS regions that encode 1,3-β-glucan synthase subunits, reducing the enzyme's sensitivity to the drug and increasing MICs.^27^ For *C. albicans*, this mechanism is led by point mutations in the FKS1 (orf19.2929) gene.^28^ Fortunately, resistance to echinocandin-class drugs remains relatively low, at <3% in *C. albicans.* Our cohort's observed decline in echinocandin resistance from 2019–2020 to 2023 is a significant finding. Several factors could explain this trend. One possible explanation is the decreased use of echinocandins following the peak of the COVID-19 pandemic.^29^ During the pandemic, widespread antifungal use, particularly in critically ill patients with COVID-19-associated candidiasis, may have contributed to selective pressure and increased resistance rates.^30^ As the pandemic subsided, hospitalisations decreased, leading to a potential reduction in antifungal exposure and a subsequent decline in resistance. Moreover, the application of an antifungal stewardship program led by ID specialists in ICUs has impacted echinocandin use and resistance.^31^

*N. glabratus* maintained alarmingly high FLU resistance rates, peaking at 85.7% in 2021 and decreasing to 55.6% in 2023. According to international literature, FLU resistance in *N. glabratus* is estimated to range between 6% and 13%.^18,32,33^ In stark contrast, our cohort exhibits an exceptionally high resistance rate, far exceeding these reported values. One of the most important virulence factors of *N. glabratus* is its intrinsic low susceptibility to azoles, with FLU MIC ∼16 times higher than that for *C. albicans.*^34,35^ This coincides with the necessity of a high dose of FLU (800 mg daily) to treat *N. glabratus* bloodstream infections.^36^ Acquired azole resistance in *Candida* species is most commonly mediated by overexpression of the ATP-binding cassette transporters CgCDR1, CgCDR2 or CgSNQ2^32,37^ and overexpression of ERG11.^32^ Unlike in other species of *Candida*, ERG11 does not appear to play a significant role in FLU resistance in *N. glabratus.*^13^ The decline in FLU resistance among *N. glabratus* isolates may be attributed to a reduction in azole consumption at our center. Lower antifungal use can decrease selective pressure, potentially limiting the emergence and persistence of resistant strains.^13^ By contrast, FLU is a fungistatic agent rather than fungicidal, so extensive use in hospital settings can allow acquired resistance.^33^ Antimicrobial stewardship programs and reduced exposure to azoles can potentially contribute to reversing or stabilising resistance trends. However, further investigation is needed to confirm this correlation and assess its long-term impact on antifungal resistance dynamics.

NAC species’ increasing prevalence and association with resistance trends demand tailored clinical responses. Given the significant burden of fungemia cases, medical wards should be prioritized for targeted interventions. These may include routine fungal surveillance, optimisation of device management, reducing NNP/TPN, and early antifungal susceptibility testing to guide therapy. Additionally, the high prevalence of *C. parapsilosis ss.* in medical wards and long-term care facilities reflects its known biofilm-forming capacity, necessitating stricter infection prevention measures. Strategies such as enhanced catheter care protocols and minimising unnecessary invasive procedures could mitigate the risk of fungemia.

This study comprehensively analyzes fungemia prevalence and antifungal resistance trends in the Venetian region; however, several limitations must be acknowledged. The absence of clinical and patient-level data significantly limits our ability to assess the underlying risk factors, sources of infection, and patient outcomes. Without information on prior hospitalisations, comorbidities, immunosuppressive conditions, or recent antifungal treatments, it is difficult to determine whether certain patient populations—such as those with hematological malignancies, neutropenia, or previous antifungal exposure—are disproportionately affected by resistant strains. Furthermore, the lack of clinical correlation prevents us from evaluating the impact of antifungal resistance on treatment efficacy, patient survival, and overall disease burden. Another limitation is the exclusive reliance on microbiological surveillance, which may underrepresent cases from outpatient settings or less-equipped healthcare facilities. Additionally, no data on the timing of blood culture positivity about patient recovery was collected, which could have provided insights into disease progression and treatment response. Future prospective studies integrating clinical, epidemiological, and outcome data are essential for a more holistic understanding of fungemia and its clinical implications.

Finally, another methodological limitation of our study is identifying *Candida* species from blood cultures using commercial standard medium vials. Detecting certain fungal species can be particularly challenging due to their slow growth and/or weak CO₂ production, as reported in several studies.^38,39^ This limitation may lead to underestimating specific *Candida* species in our dataset. To address this issue, some authors recommend using mycological blood culture vials, which enhance the likelihood of fungal isolation.^38^ However, in our study, these specialized vials were not utilized due to their unavailability, which may have impacted the detection of slow-growing or less common fungal species. As a result, the true incidence and prevalence of fungal bloodstream infections may have been underestimated, potentially leading to an underreporting of cases and an inaccurate assessment of the regional burden of fungal disease.

In conclusion, the increasing dominance of NAC species over *C. albicans*, coupled with the concentration of cases in medical wards, signifies a shift in the epidemiology of fungemia. These findings highlight the need for robust diagnostic tools, stringent infection prevention strategies, and adaptive antifungal stewardship programs to address this evolving public health challenge.
